# Diet-induced obesity promotes altered remodeling and exacerbated cardiac hypertrophy following pressure overload

**DOI:** 10.14814/phy2.12489

**Published:** 2015-08-19

**Authors:** Katherine M Holzem, Joseph T Marmerstein, Eli J Madden, Igor R Efimov

**Affiliations:** Department of Biomedical Engineering, Washington University in St. LouisSt. Louis, Missouri, USA

**Keywords:** Diet-induced obesity, electrophysiology, heart failure, hypertrophy, remodeling, signaling

## Abstract

Heart failure (HF) is the end stage of cardiovascular disease, in which hypertrophic remodeling no longer meets cardiac output demand. Established animal models of HF have provided insights into disease pathogenesis. However, these models are developed on dissimilar metabolic backgrounds from humans – patients with HF are frequently overweight or obese, whereas animal models of HF are typically lean. Thus, we aimed to develop and investigate model for cardiac hypertrophy and failure that also recapitulates the cardiometabolic state of HF in humans. We subjected mice with established diet-induced obesity (DIO) to cardiac pressure overload provoked by transverse aortic constriction (TAC). Briefly, we fed WT male mice a normal chow or high-fat diet for 10 weeks prior to sham/TAC procedures and until surgical follow-up. We then analyzed cardiac hypertrophy, mechanical function, and electrophysiology at 5–6 weeks after surgery. In DIO mice with TAC, hypertrophy and systolic dysfunction were exacerbated relative to chow TAC animals, which showed minimal remodeling with our moderate constriction intensity. Normalized heart weight was 55.8% greater and fractional shortening was 30.9% less in DIO TAC compared with chow TAC hearts. However, electrophysiologic properties were surprisingly similar between DIO sham and TAC animals. To examine molecular pathways activated by DIO and TAC, we screened prohypertrophic signaling cascades, and the exacerbated remodeling was associated with early activation of the c-Jun-N-terminal kinase (JNK1/2) signaling pathway. Thus, DIO aggravates the progression of hypertrophy and HF caused by pressure overload, which is associated with JNK1/2 signaling, and cardiometabolic state can significantly modify HF pathogenesis.

## Introduction

Heart failure (HF) is the end stage of cardiovascular diseases from numerous etiologies, in which the heart can no longer sustain the body’s metabolic requirements. The burden of HF is considerable, impacting approximately five million Americans and accounting for nearly ¼ million deaths annually (Mosterd and Hoes 2007). Thus, the translation of basic research findings into improved therapies for HF is desperately needed. To achieve this goal, many animal models of HF have been developed and thoroughly studied (Hasenfuss 1998; Houser et al. 2012). A variety of gene-specific modifications, as well as various procedures including transverse aortic constriction (TAC), infarction, and rapid pacing, have been used to provoke cardiac hypertrophic remodeling and failure. These animal models variably reflect human HF symptoms assessed by physiological parameters, such as echo- and electrocardiographic indicators, fibrosis, and natriuretic peptide levels; and the contributions of hypertrophic signaling cascades have been extensively studied.

Despite the vast knowledge we have gained from animal models of cardiac hypertrophy, the baseline phenotype of the animal prior to induction of the HF-like syndrome is generally not taken into consideration. However, cardiovascular disease, in addition to many other disorders, typically does not develop in isolation. Being overweight or obese, often due to lifestyle choices, are well-established comorbidities and/or risk factors for a variety of human diseases, including HF, several types of cancer, and stroke (Kenchaiah et al. 2002; Kress et al. 2005; Guh et al. 2009). Importantly, hypertension, a common pathologic, prohypertrophic stimulus in humans, is most often associated with being overweight or obese (Kress et al. 2005). Increased body mass and dietary metabolic substrate composition are also known to be important mediators of a variety of signaling processes (Chess and Stanley 2008; Lopaschuk and Kelly 2008; Mandavia et al. 2012), that may have significant impact on the progression of diseases. For example, c-Jun-N-terminal kinase (JNK1/2) signaling dramatically impacts metabolic regulation, and knockout of JNK1/2 isoforms abrogates the negative effects of diet-induced obesity (DIO) (Hirosumi et al. 2002; Tuncman et al. 2006; Han et al. 2013).

Previous studies have implicated diet as an important factor modulating cardiac remodeling from pressure overload (Chess et al. 2007, 2008; Duda et al. 2008; Raher et al. 2008), but these studies have not established obesity in the animals prior to HF induction. Interestingly, high-fat diet feeding without induction of insulin resistance or obesity appears potentially beneficial for cardiac remodeling; in contrast, if metabolic sequela of high-fat feeding develop, the effects are more likely detrimental. Thus, we have generated and investigated a mouse model of cardiac hypertrophy and HF, in which the animals had longstanding DIO prior to a modest pressure overload due to TAC, but did not have a genetic predisposition to HF. The DIO mice subjected to TAC had a dramatic hypertrophic response and progressed to systolic dysfunction, while the animals on a normal chow diet with TAC experienced less pronounced cardiac remodeling at the same pressure. Surprisingly, however, electrophysiologic (EP) remodeling demonstrated greater consistency in DIO compared with chow mice, regardless of surgical intervention. DIO hearts harvested following TAC, but prior to the onset of hypertrophic remodeling, demonstrate preferential upregulation of the JNK1/2 mitogen-activated protein kinase (MAPK) signaling cascade. Thus, DIO can dramatically alter the signaling processes triggering the development of cardiac hypertrophy and HF remodeling. Therefore, using models with common, comorbid diet-mediated obesity may help uncover additional insights into the basic pathologic processes of cardiovascular disease, not associated with genetic predisposition.

## Methods

### High-fat (DIO) or normal chow diet feeding and DIO phenotype assessment

**Figure 1:**
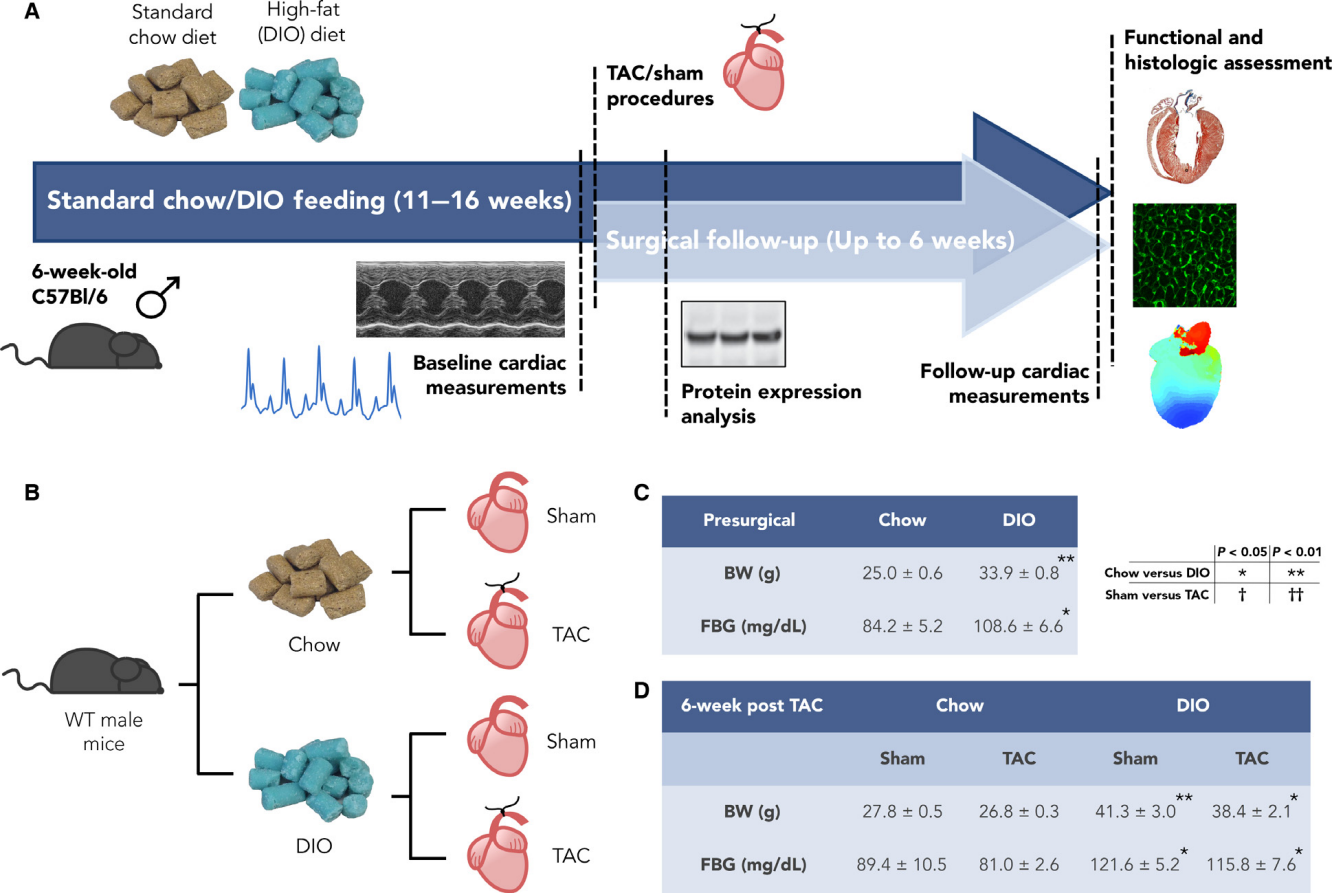
Diet-induced obesity (DIO) and pressure overload protocol and model characteristics. (A) Timeline for standard chow or high-fat (DIO) diet feeding, presurgical parameter measurement, sham/transverse aortic constriction (TAC) surgical procedures, and experimental measurements for the studies conducted. Mice were followed up at 5–6 weeks after surgery for hypertrophic and functional remodeling. For mechanistic studies, mice were followed up at 1 week after sham/TAC procedures. (B) Flowchart highlighting the experimental groupings. (C) Table of presurgical, baseline metabolic characteristics measured at 10 weeks after chow or DIO diet feeding (*n *= 13 chow; 19 TAC). Statistical significance determined with unpaired Student’s *t*-test. (D) Table of follow-up metabolic parameters, conducted at 6 weeks after surgery (*n *= 5 chow sham; 3 chow TAC; 5 DIO sham; 9 DIO TAC per group). Two-factor analysis of variance (ANOVA) was used to determine statistical significance and post hoc testing was performed with unpaired Student’s *t*-test. Data presented as mean ± SEM. BW, body weight; FBG, fasted blood glucose.

Mouse investigations were approved by the Animal Studies Committee at Washington University School of Medicine. C57BL/6J male DIO and chow diet mice were purchased from the Jackson Laboratories (Bar Harbor, ME). At 6 weeks of age, mice were initiated on a high-fat diet, containing 60% fat by kCal content (Product D12492, Research Diets, New Brunswick, NJ), to induce obesity, or were maintained on a standard chow diet, containing 13–16% fat by kCal content, and mice were continued on their respective diets for total of 11 or 16 weeks. The mice had ad libitum access to food and water for 10 weeks before undergoing baseline studies and TAC/sham procedures. High-fat or standard chow diet feeding was maintained after surgery for 1 week for protein expression analysis or 6 weeks for heart mass and function studies. See Figure1A, B for protocol timeline and experimental groupings. For metabolic phenotype evaluation, mice were weighed and had 6-h fasted blood glucose (FBG) measurements taken at 16 and 22 weeks of age (Fig.1C). Blood samples were collected via lateral saphenous vein bleed, and a standard FreeStyle Lite (Abbott Laboratories, Abbott Park, IL) glucometer was used to obtain FBG concentrations.

### TAC or sham surgery

**Figure 2:**
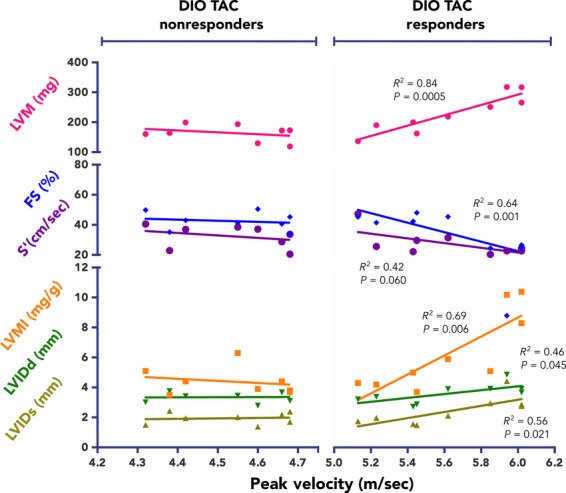
Peak velocity correlates with response only for high afterload pressures in diet-induced obesity (DIO), transverse aortic constriction (TAC) mice. Postsurgical echocardiographic measurements were collected at 5 weeks post-TAC. DIO TAC mice having a PV at the aortic band ≤4.75 m/sec demonstrate no relationship to degree of band tightness. No regression lines diverge significantly from zero, and goodness-of-fit values are low (*n *= 8). In contrast, there are strong correlations between several electrocardiographic response measures and PV when PV exceeded 4.75 m/sec. Most parameters have regression lines that strongly differ from zero slope and have high goodness-of-fit values (*n *=9). Linear regression analysis was performed by minimizing the vertical SS distance. *P* and *R*^2^ values displayed on graph. FS, fractional shortening; LV, left ventricle; LVIDd, LV internal dimension – diastole; LVIDs, LV internal dimension – systole; LVM, LV mass; LVMI, LV mass index; SS, sum-of-squares; S’, peak velocity of the systolic annulus.

The protocol for aortic constriction was based on the original description by Rockman et al. (1991, 1994). Mice were anesthetized with ketamine (100 mg/kg) and xylazine (10 mg/kg) and were intubated. Following blunt dissection, the aorta was identified and freed, and a 7.0 silk suture was tied around both the transverse aorta and a 27-gauge blunt needle. Needle gauge and, thus, band tightness were determined based average body weight, and the same gauge was used for all TAC animals in the study. Sham-operated mice underwent the same operation, without suture tied around the aorta. For analysis, we included only mice having a peak velocity (PV) of blood flow at the aortic band consistent with sufficient pressure to potentially induce hypertrophic remodeling. The PV threshold was set at the cut-off between the region where echocardiography parameters demonstrated no correlation with PV and where there was a strong, linear dose dependence of the TAC manipulation. This dose dependence started at PV = 4.75 m/sec (Fig.2), and thus this was the cut-off threshold for inclusion for chow and DIO TAC mice. However, several response measures maintained statistical significance for DIO TAC compared with chow TAC hearts, even when the data were diluted by animals with insufficient PV (data not shown).

### Echocardiography

Noninvasive ultrasound examination of the cardiovascular system was performed using a Vevo Ultrasound System (VisualSonics Incorporated, Toronto, Ontario, Canada). First, mice were lightly anesthetized with 2% Avertin and then switched to continuous inhalation with 1.5% gaseous isoflurane. Ultrasound studies were performed using multiple transducers with different ultrasound frequencies (30–60 MHz) depending on the size and depth of the cardiovascular structure of interest. Two-dimensional and M-mode images were collected for baseline LV measurements, and complete two-dimensional, M-mode, and Doppler ultrasound examination was performed for postsurgical follow-up measurements.

### Optical imaging

Mice were heparinized and anesthetized with ketamine (100 mg/kg) and xylazine (10 mg/kg). Then, hearts were excised and cannulated through the aorta via Langendorff methods. Hearts were perfused with 37°C, oxygenated Tyrode’s solution, maintained at a perfusion pressure of 60–80 mmHg, and pinned with the posterior surface facing toward the optical apparatus. We then immobilized hearts with the excitation–contraction uncoupler, blebbistatin (10–20 *μ*mol/L), and stained with the voltage sensitive dye, Rh-237 (10 mmol/L), as in previous reports (Dou et al. 2007; Glukhov et al. 2010; Lang et al. 2011). Pacing was conducted using an S1S1 steady-state restitution protocol starting at 150–200 msec and decreasing to the functional refractory period, with an Ag/AgCl electrode placed near the cardiac apex. Fluorescence data were acquired with a MiCAM Ultima-L CMOS camera system and displayed with Brainvision Software (SciMedia Ltd., Costa Mesa, CA). Custom-written MATLAB software was used to calculate conduction velocity (CV) and action potential duration at 80% repolarization (APD80) for the posterior LV apex (Laughner et al. 2012).

### Histology and immunohistochemistry

Isolated mouse hearts were fixed in 10% formalin. Hearts were dehydrated and paraffin embedded. Sections were cut at 10 *μ*m thickness. Masson’s Trichrome (MT) and wheat germ agglutinin (WGA) stainings were carried out using standard methods. Immunohistochemical staining was conducted with an anti-Mac-2 antibody (Cedarlane, Burlington, NC), and slides were counterstained with Harris hematoxilin. Images were acquired using Leica MZ8 (Leica Microsystems, Buffalo Grove, IL), EVOS XL Core (Life Technologies, Grand Island, NY), Nikon D-Eclipse C1 (Nikon Instruments, Melville, NY), or NanoZoomer (Hamamatsu, Hamamatsu City, Japan) microscopes, and analysis was performed using ImageJ software.

### Protein expression and kinase activation analysis

Prior to heart recovery, mice continued to feed ad libitum. Following recovery, isolated intact mouse hearts were Langendorff perfused with ice-cold cardioplegic solution. The anterior LV was then dissected and prepared for protein expression analysis. Briefly, protein lysates were prepared by pulverization in liquid nitrogen and homogenization in super RIPA buffer. Protein was quantified using the BCA Protein Assay (Thermo Scientific, Buffalo Grove, IL), and equal masses were loaded into each lane. SDS-PAGE was carried out using standard methods. Blots were then probed with specific anti-kinase or anti-phospho-kinase antibodies (Cell Signaling Technologies, Boston, MA) in bovine serum albumin (BSA) blocking buffer. Images were acquired using a LAS-4000 mini, and quantification was performed using MultiGauge software (Fujifilm, Tokyo, Japan).

### Statistical analysis

Data are presented as mean ± SEM. Two-factor analysis of variance (ANOVA; diet and surgical intervention) was used to determine statistical significance, with the Type III sum-of-squares (SS) reported to account for the unbalanced design. Where appropriate, post hoc testing was conducted using unpaired Student’s *t*-test with two-tailed distribution. Grubb’s analysis was used to exclude outliers. Linear regression analysis was performed by minimizing the vertical SS distance from data points to the line. *R*^2^ values were computed by the equation *R*^2^ = 1 – (SS_reg_/SS_tot_), where SS_reg_ = SS for regression line and SS_tot_ = SS for horizontal line through the *y*-axis mean. GraphPad Prism software was used for statistical analyses. *P *<* *0.05 was considered statistically significant.

## Results

### Protocol for pressure overload with DIO

We have developed a protocol for induction of hypertrophic remodeling and HF in a mouse on a DIO background. Figure1A details the feeding and pressure overload induction timeline for the animals utilized in this study, and the flowchart in Figure1B highlights the experimental groups for study. At 6 weeks of age, WT male C57BL/6J mice were randomized to receive ad libitum feeding with either a standard chow diet, containing 13–16% fat by kCal content, or a high-fat diet, containing 60% fat by kCal content, and the two groups of animals were maintained on these diets for 10 weeks before pressure overload with TAC. For studies of the hypertrophic response, the animals were then continued on their respective diets for an additional 6 weeks during the development of hypertrophy.

Body weight (BW) and 6-h FBG measurements were taken prior to the surgical procedures and at the end of the protocol to evaluate the metabolic phenotype in our model and to assess the impact of the surgical manipulation on these measures. Figure1C shows presurgical parameters after 10 weeks of diet for chow and DIO animals, and Figure1D highlights the BW and FBG measurements for the four experimental groups at 6 weeks postsurgical intervention. The DIO mice have significantly greater average BW than the standard chow groups at both time points, and BW and FBG measurements of the TAC animals at 6 weeks after surgery are not significantly different from sham-operated animals. Thus, in our model, the mice have established DIO and hyperglycemia prior to TAC/sham operation, and this phenotype is maintained several weeks after surgery.

### Dose-dependence of pressure overload for mice with DIO

When analyzing the response to surgery in our mice, we clearly observed minimal response to TAC for any of the normal weight, chow-diet animals, and, thus, there were no significant correlations between output measures and PV for chow TAC animals. However, the hypertrophic and functional response for DIO TAC animals appeared graded in magnitude. We hypothesized that the degree of the phenotypic response for DIO mice was due to the TAC banding pressure applied within the range of PVs demonstrated in our study. With linear correlation analysis, we demonstrated that there is no relationship of PV to many of the echocardiographic measures if the PV ≤ 4.75 m/sec; thus, we categorized all TAC animals with PV ≤ 4.75 m/sec as nonresponders (Fig.2). In contrast, with PV > 4.75 m/sec, there were strong linear correlations between PV and several of the measured echocardiographic parameters for DIO mice. This indicates dose dependency of TAC pressure, and is a main criterion for causal effect of a biological manipulation (Hill 1965). Thus, in our analysis we only considered animals with a great enough pressure overload stimulus to potentially have an effect of interest for analysis. Importantly, the degree of TAC appeared to be the main factor influencing the response to surgical manipulation, as there was no relationship of presurgical BW to the degree response (data not shown).

### Cardiac hypertrophy exacerbated with DIO and TAC

**Figure 3:**
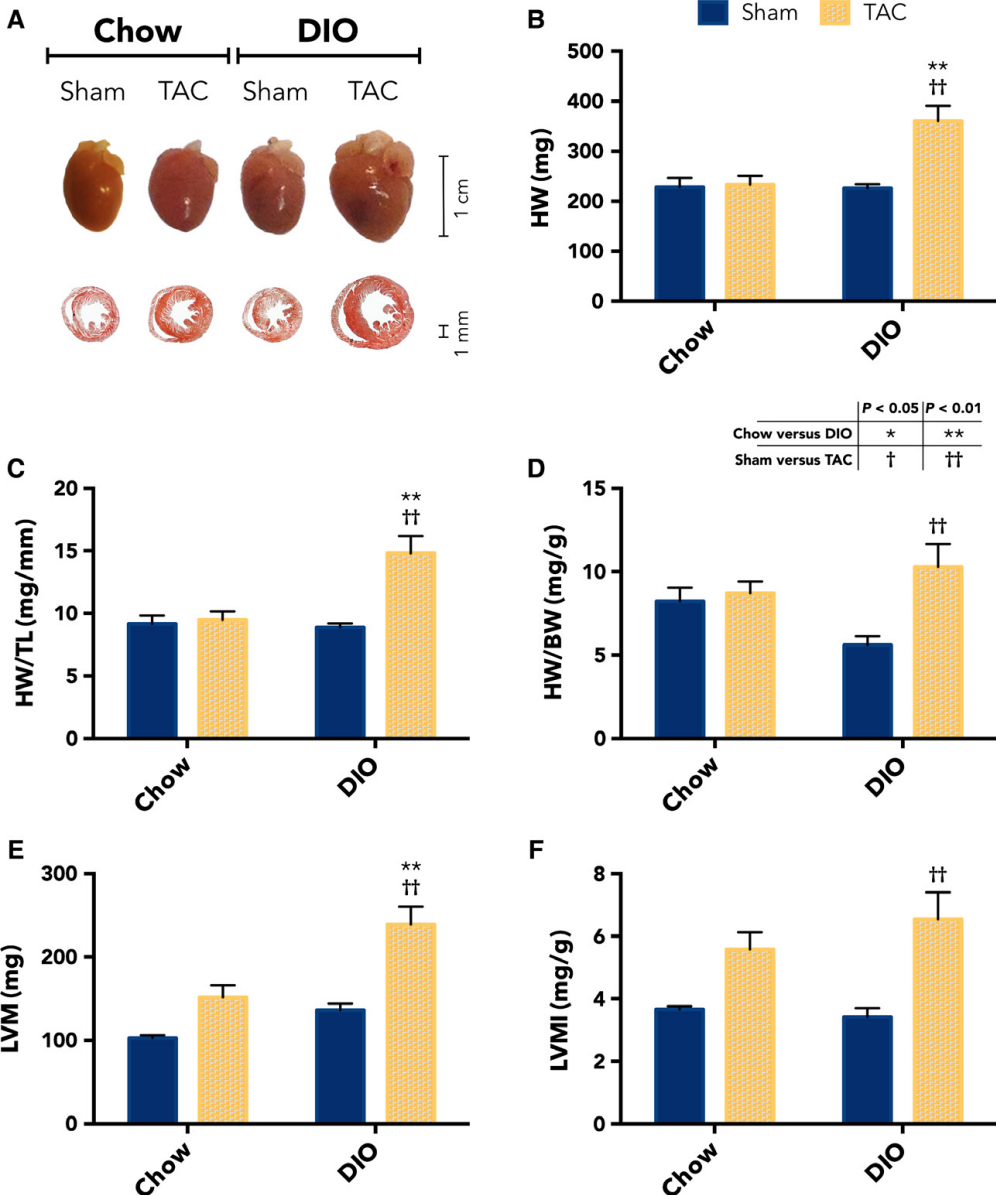
Hypertrophic response is dramatically potentiated in transverse aortic constriction (TAC) mice with diet-induced obesity (DIO). (A) Representative images of formalin-fixed whole mouse hearts and Masson’s trichrome stained transverse heart sections from each experimental group. (B) Raw HW and (C) normalized HW/TL measured after heart excision at 6 weeks postintervention are significantly greater for the DIO TAC compared with chow TAC hearts (*n *= 5 chow sham; 3 chow TAC; 5 DIO sham; 7 DIO TAC). (D) HW/BW ratio is not different between DIO TAC and chow TAC hearts, due to increase the in BW denominator for DIO mice (*n *= 5 chow sham; 3 chow TAC; 5 DIO sham; 7 DIO TAC). (E) LVM, measured during echocardiography at 5-week follow-up, is greater for DIO TAC compared with chow TAC hearts (*n *= 5 chow sham; 4 chow TAC; 7 DIO sham; 10 DIO TAC). F. LVMI was not different between groups. Data presented as mean ± SEM. Two-factor analysis of variance (ANOVA) was used to determine statistical significance and post hoc testing was performed with unpaired Student’s *t*-test. BW, body weight; HW, heart weight; LVM, LV mass; LVMI, LV mass index; TL, tibia length.

The cardiac hypertrophic response to pressure overload is dramatically increased on a background of DIO compared with animals fed on a chow diet. Figure3A shows representative images of formalin-fixed whole mouse hearts and MT stained heart sections. Raw wet heart weights (HWs) are much greater for the DIO TAC hearts than for all other groups, including the chow TAC hearts, which did not increase in weight at the TAC banding tightness used in this study (36.0 ± 3.1 vs. 23.3 ± 1.8 mg, *P* = 0.004, *n* = 3–7 per group; Fig.3B). Figure3C, D demonstrates HW normalized to tibia length (TL) and to BW. The HW/TL ratio is 55.8% greater for the DIO TAC group when compared with chow TAC mice (1.48 ± 0.14 vs. 0.95 ± 0.07 mg/mm, *P* = 0.006, *n* = 3–7 per group; Fig.3C). However, the HW/BW ratio for DIO TAC mice is not significantly greater than other groups, due to relative greater increase in BW for DIO mice (1.03 ± 0.14 vs. 0.87 ± 0.07 mg/mm, *P* = 0.37, *n* = 3–7 per group, for DIO TAC vs. chow TAC; Fig.3D). This is also demonstrated by reduced trend in HW/BW for DIO sham mice relative to chow sham animals (0.56 ± 0.05 vs. 0.82 ± 0.08 mg/g, *P* = 0.11, *n* = 3–7 per group; Fig.3D).

Echocardiographic measures of left ventricular (LV) mass (LVM) also demonstrate hypertrophy in DIO TAC relative to chow TAC hearts. Interestingly, LVM is increased by 57.8% in the DIO TAC hearts relative to the chow TAC hearts (239.2 ± 21.1 vs. 151.6 ± 14.8 mg, *P* = 0.004, *n* = 4–10 per group; Fig.3E), which is similar in degree to the increase for normalized HW/TL. In contrast, LV mass index (LVMI) is not significantly different for DIO TAC hearts relative to the chow TAC hearts (6.55 ± 0.86 vs. 5.58 ± 0.55 mg/g, *P* = 0.38, *n* = 4–10 per group; Fig.3F), because the BW increase masks the difference in LVM.

**Figure 4:**
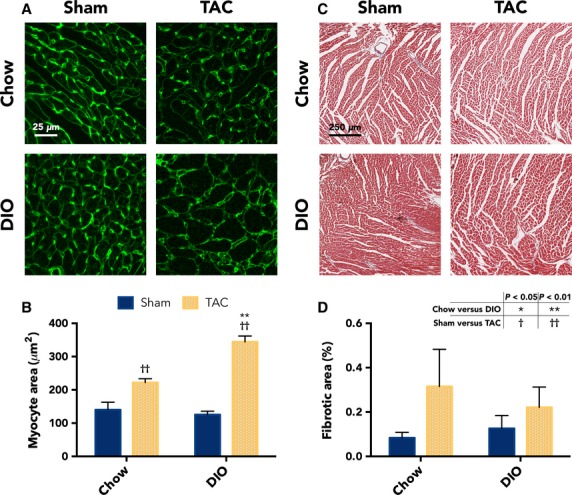
Remodeling from diet-induced obesity (DIO) and transverse aortic constriction (TAC) due to cardiomyocyte hypertrophy, and fibrosis not increased in DIO TAC hearts. (A) Representative images from WGA-stained heart sections with short-axis cardiac myocyte views from hearts recovered 6 weeks after surgical intervention. (B) Average cardiomyocyte area quantified from WGA images showing that myocytes from DIO TAC hearts are significantly larger than chow TAC myocytes (*n *= 3 per group). (C) Representative MT stained sections imaged at 10x magnification from hearts recovered at 6 weeks after surgical intervention. (D) Quantitative analysis of LV free wall fibrotic area (blue staining) expressed as a percent of total tissue area. There are no significant differences among the means of any groups, due to the overall low proportion of fibrosis and the large variance within groups. In addition, there is no greater fibrosis in DIO TAC compared with chow TAC hearts (*n *= 3 per group). Data presented as mean ± SEM. Two-factor analysis of variance (ANOVA) was used to determine statistical significance and post hoc testing was performed with unpaired Student’s *t*-test. MT, Masson’s trichrome; WGA, wheat germ agglutinin.

We then examined whether the increased size of DIO TAC hearts was due to cardiomyocyte hypertrophy by measuring myocyte cross-sectional area. We stained short-axis heart sections with WGA, which labels cell membranes, and Figure4A shows representative WGA images from each group. We then quantified number of pixels within cardiomyocyte boundaries, which revealed that DIO TAC cardiomyocytes are 55.0% greater in size compared with chow TAC myocytes (344.6 ± 17.3 vs. 222.3 ± 11.4 *μ*m^2^, *P* = 0.0007, *n* = 3 per group; Fig.4B). In addition, fibrotic area of the LV free wall, quantified from MT staining, was not significantly greater in DIO TAC hearts compared with other groups (Fig.4C, D).

### DIO with TAC worsens systolic function

In addition to having a dramatic hypertrophic response, the hearts from the DIO mice with TAC also develop greater systolic dysfunction than the chow TAC mice. Table1 shows echocardiographic parameters collected at 5 weeks following TAC/sham surgery. From these echocardiographic measures, the LV posterior wall (LVPW) thickness is 22.6% greater in the DIO TAC hearts compared with the chow TAC hearts during diastole and approaches significance during systole (1.84 ± 0.09 vs. 1.60 ± 0.06 mm, *P* = 0.053, 4–10 per group; Table1).

**Table 1:** Summary of follow-up echocardiographic parameters

Echo parameters	Chow Sham (*n *= 5)	Chow TAC (*n *= 4)	DIO Sham (*n *= 7)	DIO TAC (*n *= 10)
IVSd (mm)	0.95 ± 0.02	1.23 ± 0.05††	1.09 ± 0.09	1.42 ± 0.07††
LVIDd (mm)	3.21 ± 0.11	3.22 ± 0.12	3.28 ± 0.24	3.69 ± 0.19
LVPWd (mm)	0.94 ± 0.03	1.15 ± 0.06	1.14 ± 0.11*	1.41 ± 0.06*,††
IVSs (mm)	1.40 ± 0.02	1.74 ± 0.02†	1.62 ± 0.10	1.84 ± 0.09
LVIDs (mm)	1.77 ± 0.11	1.69 ± 0.10	1.82 ± 0.11	2.55 ± 0.34
LVPWs (mm)	1.42 ± 0.03	1.60 ± 0.06	1.59 ± 0.10	1.84 ± 0.09†
IVCT (msec)	4.70 ± 0.36	5.17 ± 0.40	5.96 ± 0.23	7.16 ± 0.77*
IVRT (msec)	9.82 ± 0.54	14.64 ± 1.08††	10.98 ± 0.57	13.02 ± 0.73†
FS (%)	45.00 ± 2.18	47.52 ± 2.11	44.72 ± 0.90	32.85 ± 5.09*
VTI ratio	1.00 ± 0.07	4.89 ± 0.29††	0.94 ± 0.07	5.56 ± 0.41††
PV (m/sec)	1.56 ± 0.06	5.68 ± 0.39††	1.35 ± 0.14	5.55 ± 0.22††

Data presented as mean ± SEM. IVSd, interventricular septum – diastole; LVIDd, LV internal dimension – diastole; LVPWd, LV posterior wall – diastole; IVSs, interventricular septum – systole; LVIDs, LV internal dimension – systole; LVPWs, LV posterior wall – systole; IVCT, isovolumic contraction time; IVRT, isovolumic relaxation time; FS, fractional shortening; VTI Ratio, velocity–time integral ratio; PV, peak velocity.

Measurements from echocardiographic studies collected at 5 weeks after surgical intervention. Two-factor ANOVA was used to determine statistical significance and post hoc testing was performed with unpaired Student’s *t*-test.

* *P* < 0.5

** *P *< 0.01 chow versus DIO.

† *P *< 0.05

†† *P *< 0.01 sham versus TAC.

In addition, isovolumic contraction time (IVCT) is increased 1.38-fold in the DIO TAC relative to chow TAC hearts, while isovolumic relaxation time (IVRT) is not different between TAC groups. Percent fractional shortening (FS) of the LV is also reduced by 30.9% in the DIO TAC hearts relative to the chow TAC hearts (*P* = 0.015, 4–10 per group; Table1). Importantly, the TAC banding was not different between the groups subjected to TAC, and this is indicated in Table1 by velocity–time integral (VTI) ratio and PV of blood flow at the aortic band, which are not different between DIO and chow TAC groups. Taken together, these results indicate systolic, but not diastolic, dysfunction in mice with both obesity and pressure overload.

### Electrical remodeling trends with metabolic status

**Figure 5:**
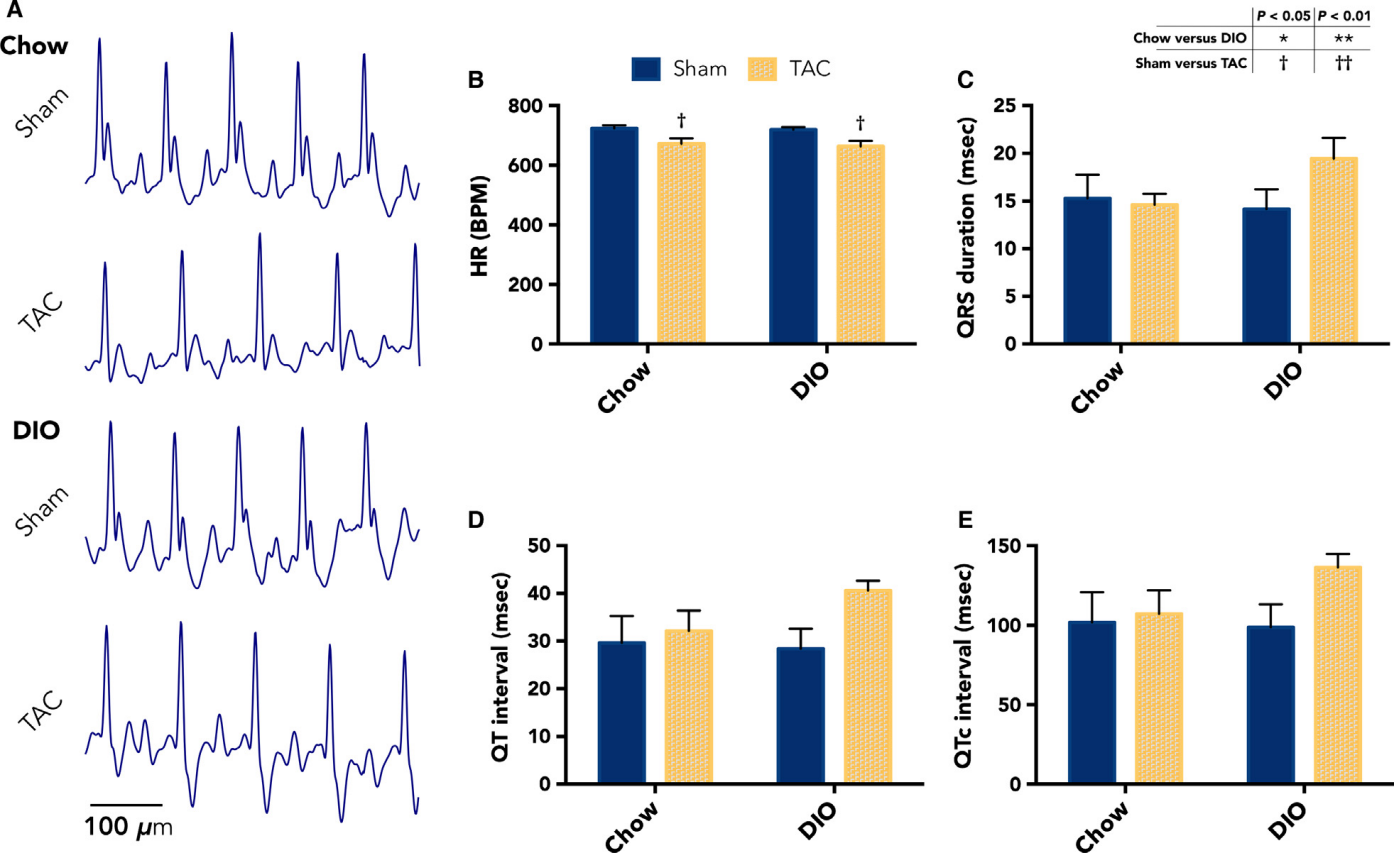
Lack of electrocardiographic alterations in diet-induced obesity (DIO) mice with transverse aortic constriction (TAC). (A) Representative, in vivo, unanesthatized ECG traces collected from mice in each experimental group at 6-week surgical follow-up. (B) Graph showing no significant differences in average heart rate between DIO TAC and chow TAC animals. (C) Average QRS durations show an increased trend for DIO TAC versus chow TAC mice, though not significant. Although there is an increased trend for DIO mice with TAC, (D) QT and (E) QTc intervals are also not different for DIO TAC compared with chow TAC mice (*n *= 5 chow sham; 4 chow TAC; 5 DIO sham; 7 DIO TAC). Data presented as mean ± SEM. Two-factor analysis of variance (ANOVA) was used to determine statistical significance and post hoc testing was performed with unpaired Student’s *t*-test. BPM, beats per minute.

**Figure 6:**
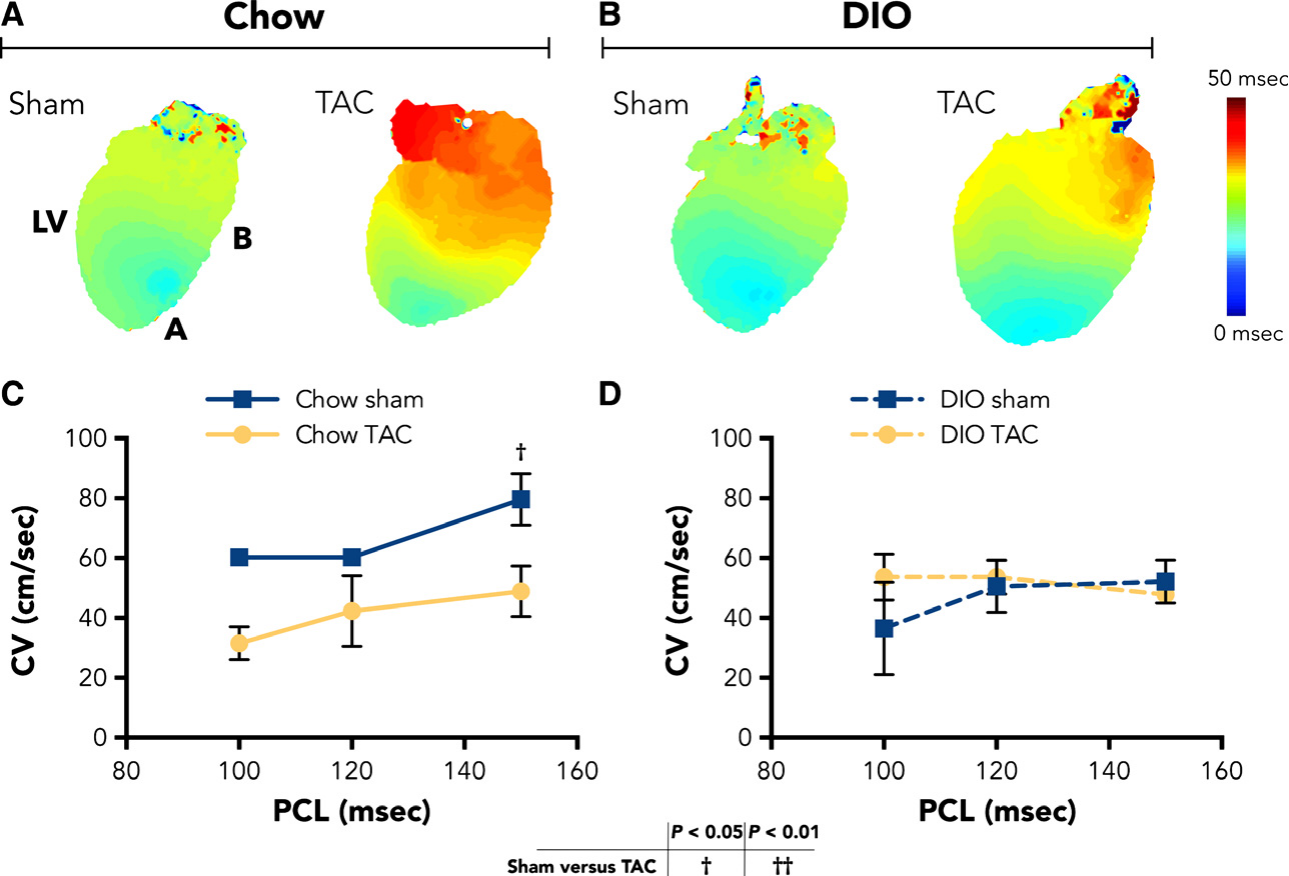
CV measurements are similar for diet-induced obesity (DIO) animals. Ex vivo CV parameters were collected at 6 weeks after surgical intervention. Representative activation maps from (A) chow and (B) DIO hearts at 150 msec PCL demonstrating that CV is reduced in chow TAC but not DIO TAC hearts. (C) CV restitution curves for chow hearts, showing slowing of CV with TAC. (D) CV restitution curves for DIO hearts demonstrating overlapping curves for DIO sham and DIO TAC hearts (*n *= 3 chow sham; 3 chow TAC; 5 DIO sham; 6 DIO TAC). Data presented as mean ± SEM. Two-factor analysis of variance (ANOVA) was used to determine statistical significance and post hoc testing was performed with unpaired Student’s *t*-test. CV, conduction velocity; PCL, pacing cycle length.

Although there is dramatic hypertrophic remodeling with DIO and TAC, we found that EP properties appeared to associate more with animal metabolic state, regardless of surgical manipulation. We first examined in vivo, unanesthetized ECG parameters at 6-week surgical follow-up (representative traces in Fig.5A) and found no differences in HR, QRS duration, or QT interval for DIO TAC compared with Chow TAC animals (Fig.5B–E). Given the degree of hypertrophic remodeling, we further characterized EP alterations by whole-heart optical imaging. Figure6A, B show representative activation maps for chow and DIO hearts at 150 msec pacing cycle length (PCL), showing similar conduction for both DIO sham and TAC hearts. In addition, while ventricular conduction is slowed with TAC, it appears better preserved in DIO TAC compared with chow TAC hearts. We then measured CV at several PCLs and plotted restitution curves (Fig.6C, D). We found that TAC reduced the CV of chow heart LVs (79.6 ± 8.6 vs. 48.9 ± 8.4 cm/s at 150 ms PCL, *P* = 0.01); however, the CVs were not different for DIO sham and DIO TAC hearts at each PCL.

**Figure 7:**
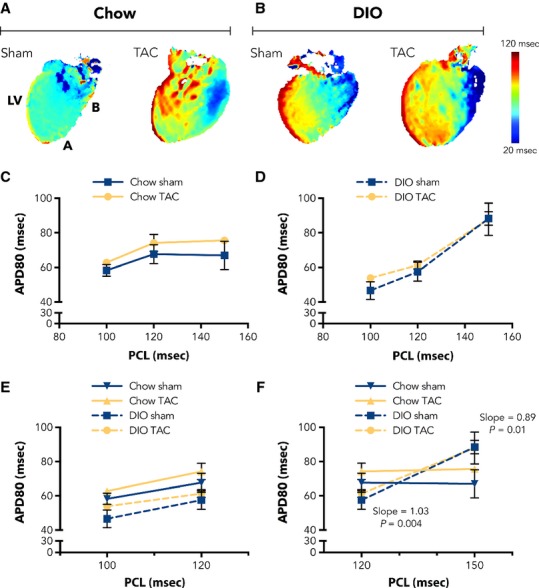
APD80 values trend with metabolic state. Ex vivo APD80 parameters were collected at 6 weeks after surgical intervention. Representative APD80 maps from (A) chow and (B) diet-induced obesity (DIO) hearts at 150 msec PCL. APD80 restitution at curves for (C) chow and (D) in DIO hearts at several PCLs, highlighting the apparent similarities between APD80 curves based on dietary treatment. (E) APD80 values and best-fit regression lines between 100 and 120 msec PCLs. Note that regressions for all heart groups have similar slopes in this pacing frequency region. (F) APD80 values and regression lines between 120 and 150 msec PCLs. Both chow groups have similar regressions, with slopes that do not differ from zero. Also, both DIO groups have regression slopes that approximate one and are significantly different from zero. APD80 = action potential duration at 80% repolarization (*n *= 3 chow sham; 3 chow TAC; 5 DIO sham; 6 DIO TAC). Data presented as mean ± SEM. Two-factor analysis of variance (ANOVA) was used to determine statistical significance. Linear regression analysis was performed by minimizing the vertical SS distance.

Likewise, LV APD80 values trended with metabolic state of the animals. Representative APD80 maps are displayed in Figure7A, B, highlighting similarities between hearts from mice on the same diets. Restitution curves for APD80 values at several PCLs are plotted in Figure7C, D for chow and DIO hearts, showing similarity between curve slopes. For additional precision, we then separately plotted APD80 values for 100–120 msec and 120–150 msec, with associated linear regressions (Fig.7E, F). All APD80 linear regressions between 100 and 120 msec demonstrate similar slopes. In contrast, linear regressions between 120 and 150 msec are different for chow and DIO hearts. Slopes for chow sham and chow TAC hearts are −0.02 ± 0.33 (*P* = 0.95) and 0.05 ± 0.17 (*P* = 0.78), respectively, indicating flat regressions for both chow groups. However, DIO sham and DIO TAC linear regression slopes are 1.03 ± 0.22 (*P* = 0.004) and 0.89 ± 0.26 (*P* = 0.01), respectively. Thus, DIO sham and TAC hearts have similarly steep regressions in this pacing frequency range. These data suggest that cardiac EP properties may critically associate with cardiometabolic state, despite additional cardiovascular insult.

### JNK1/2 signaling activated selectively with DIO and TAC

**Figure 8:**
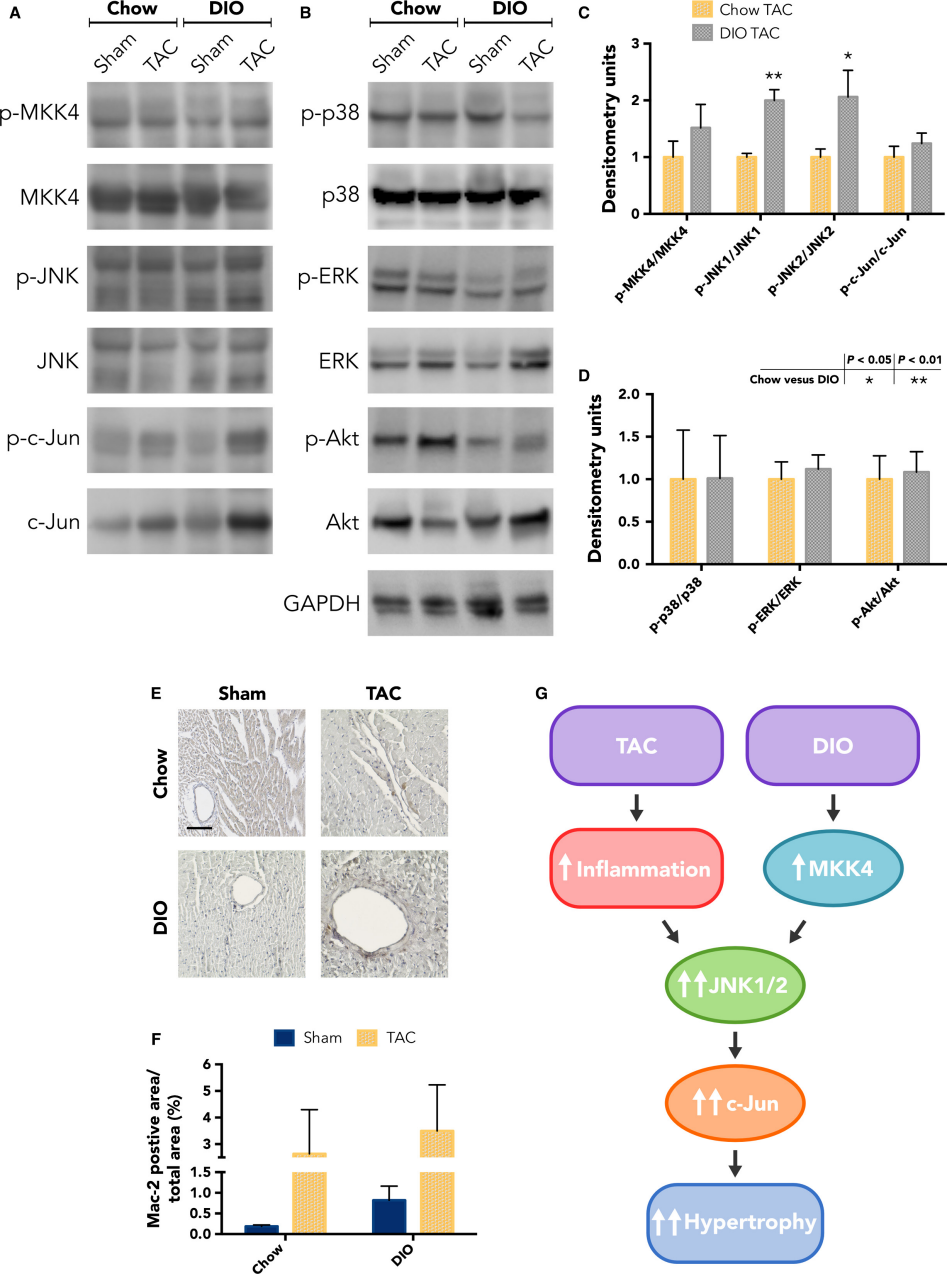
The JNK1/2 signaling pathway is activated selectively with diet-induced obesity (DIO) and transverse aortic constriction (TAC). (A) Representative Western blot images for JNK1/2 pathway signaling kinases and their phosphorylated isoforms examined at 1 week postsurgical intervention. (B) Representative Western blot images for several additional prohypertrophic signaling kinases and their phosphorylated isoforms. (C) Densitometric quantification of phospho-kinase/kinase ratios for the JNK1/2 signaling pathway, showing activation with DIO and TAC (*n *= 6 chow sham; 6 chow TAC; 4 DIO sham; 7 DIO TAC). (D) Phospho-kinase/kinase density ratios for additional signaling kinases, indicating no difference in activation between DIO TAC and chow TAC LV samples (*n *= 6 chow sham; 6 chow TAC; 4 DIO sham; 7 DIO TAC). (E) Representative 20x immunohistochemistry images from each group, with Mac-2 labeling indicated by brown staining. Scale bar = 100 *μ*m. (F) Quantification of Mac-2 positive area relative to total tissue area for the LV free wall. Although there is high variability in TAC groups, there appear to be trends toward significance for TAC relative to sham hearts (*n *= 3 chow sham; 3 chow TAC; 2 DIO sham; 3 DIO TAC). Data presented as mean ± SEM. Two-factor analysis of variance (ANOVA) was used to determine statistical significance and post hoc testing was performed with unpaired Student’s *t*-test.

To examine the mechanism promoting cardiac hypertrophy and dysfunction in mice with DIO and TAC, we assessed several of the known hypertrophy-inducing signaling cascades at 1 week after TAC. Surprisingly, we did not find increased activity of several prohypertrophic cascades, including p38 MAPK, extracellular signal-regulated kinase (ERK1/2), or Akt, as assessed by the levels of phosphorylated kinase relative to total kinase density (Fig.8B, D). Instead, we found that the JNK1/2 signaling pathway was selectively upregulated in the LV from hearts with both DIO and TAC (Fig.8A, C). We observed a 2.00-fold increase in p-JNK1/JNK1 density, *P* = 0.0002 (*n* = 4–7 per group), and a 2.06-fold increase in p-JNK2/JNK2, *P* = 0.02 (*n* = 4–7 per group), for chow TAC versus DIO TAC LV samples (Fig.8C). We then proceeded to screen upstream and downstream members of the JNK pathway and found that additional pathway components, p-MKK4/MKK4 and p-c-Jun/c-Jun, trended toward activation with DIO and TAC. In addition to increased c-Jun activation, we also observed increased total c-Jun protein expression with TAC (data not shown), suggesting additional translational or posttranslational regulation c-Jun and minimizing the apparent activated c-Jun (p-c-Jun/c-Jun).

Because inflammation is thought to be an important mediator of hypertrophy and metabolic responses, we also analyzed macrophage infiltration into the myocardium at 1 week after TAC. We conducted immunohistochemistry on tissue sections with antibodies to the macrophage-specific marker, Mac-2, which coincidentally, is also an important biomarker for HF prognosis in patients.(Felker et al. 2012) Representative 20x Mac-2 immunohistochemistry images, with specific labeling indicated by brown staining, are shown in Figure8E. After quantifying the Mac-2 positive area over total tissue area for the LV free wall, we found a trend toward greater staining in both TAC groups, with no difference between DIO TAC and chow TAC (Fig.8F). These results suggest that inflammation occurs as a result of TAC, regardless of initial body habitus. We summarized our signaling results with a schematic in Figure8G, highlighting the proposed synergy of the JNK pathway response to combined DIO and TAC.

## Discussion

These findings indicate that high-fat DIO is a powerful promoter of hypertrophic remodeling in the heart when combined with a pressure overload stimulus, even in the absence of genetic predisposition. Standard chow diet fed, nonobese mice develop little cardiac hypertrophy in response to moderate TAC within the range of pressures applied; however, the cardiac hypertrophic response is much greater with TAC in mice with a DIO background. Our findings indicate that there is a synergistic effect of obesity and pressure overload for cardiac hypertrophy, as neither stimulus alone was sufficient to induce substantial remodeling in this study. Importantly, we also demonstrate a dose-dependent response to the degree of TAC banding pressure applied for the DIO mice. This suggests that our TAC intervention is both causal of the hypertrophic remodeling and that DIO mice exhibit a response to afterload starting at a lower pressure range than that which causes remodeling in normal weight animals. Interestingly, the increase in heart mass in DIO TAC hearts appears primarily due to increased LV cardiomyocyte volume. This is demonstrated by the similarly scaled increases in HW/TL, LVM, and cardiomyocyte volume for DIO TAC compared with chow TAC hearts. We also examined fibrotic area and found no difference between the two TAC groups (Fig.5).

Although it may seem unexpected that our chow TAC animals did not have substantial hypertrophy, this is because the band tightness (needle gauge) was determined based on the average weight of all animals in the study. Because there were many DIO mice, a relatively wide needle was used to set constriction intensity. The resulting afterload pressure from the larger needle was not sufficient to induce hypertrophy in chow animals. Although, at first, this may seem a limitation of the study, we actually consider this to be a critical strength of this investigation. We have demonstrated that there is a range of afterload pressure insufficient to induce significant hypertrophy in chow diet animals, but that same pressure range can produce tremendous hypertrophy in DIO mice.

### Hypertrophic signaling with DIO and TAC

In several HF models, pathologic, prohypertrophic signaling pathways are activated, including arms of the MAPK pathway. Isoforms of p38 MAPK, most notably *α* and *β*, have been implicated in hypertrophic remodeling and cardiomyopathy (Marber et al. 2011). Overexpression of the upstream p38 MAPK kinase (MKK) activators produces cardiomyopathy. Similarly, unrestrained activation of p38 leads to cardiac dysfunction (Wilkins et al. 2004; Auger-Messier et al. 2013). In addition, the ERK1/2 pathway promotes pathologic hypertrophy – constitutively active ERK1/2 leads to hypertrophy and cardiomyopathy, while ERK1/2 downregulation mitigates hypertrophic remodeling (Rose et al. 2010).

In contrast, physiologic hypertrophy is critically dependent on insulin or insulin-like growth factor (IGF) signaling through Akt (McMullen et al. 2003; DeBosch et al. 2006; Walsh 2006; Kemi et al. 2008). Insulin binds to insulin or IGF receptors and initiates downstream hypertrophic signaling, making Akt a critical regulator of growth from nutritional input (Shiojima et al. 2002; Walsh 2006). With metabolic disease, such as type 2 diabetes, the myocardium maintains insulin sensitivity while systemic insulin resistance develops, and consequent hyperinsulinemia can potentiate cardiac Akt signaling (Jagasia et al. 2001). This becomes particularly important because constitutive, instead of intermittent, activity of Akt can cause pathologic hypertrophy (Chu et al. 2009). In addition, pressure overload has been shown to upregulate cardiac insulin receptor expression and hypertrophy through enhanced mechanical stretch, and plasma insulin depletion attenuates this response (Shimizu et al. 2010).

Alternatively, the role of JNK1/2 isoforms in cardiac hypertrophic remodeling has been less clear. JNK1/2 signaling is upregulated following pressure overload, and overactivation of the upstream JNK1/2 activator, MKK7, leads to a hypertrophic phenotype (Wang et al. 1998; Rose et al. 2010). Conversely, reduction in the MKK4 activator has either abolished or increased the hypertrophic response to pressure overload (Choukroun et al. 1999; Liu et al. 2009; Rose et al. 2010). Additional work has suggested differential activation of the various MAPK pathways based on stimulus type and intensity, that is, JNK1/2 and ERK1/2 appeared more activated with strong pressure overload, whereas p38 MAPKs were triggered by volume overload (Sopontammarak et al. 2005). Differential pathway activation may be due to condition-dependent G protein-coupled receptor (GPCR) stimulation (Esposito et al. 2001). Taken together, signaling kinases, including the MAPKs, are important regulators of cardiac hypertrophy; however, different pathways may be activated by distinct prohypertrophic stimuli. In contrast to previous work, we did not observe increased activation of Akt, the primary effector of insulin signaling, following TAC. Our results suggest that an alternative pathway, through JNK1/2, is mediating the increase in hypertrophy. Thus, our results are distinct from the work of Raher et al. (2008), as they induce systemic insulin resistance, but not obesity in their mice prior to pressure overload. Additionally Steinbusch et al. (2011), study the effects of cluster of differentiation 36 (CD36) knockout (KO) on the response to a Western-type diet. While they similarly observe a greater hypertrophic and functional response with long-term Western-type diet feeding and TAC, their result may be due to systemic insulin insensitivity. CD36 KO not only improves the phenotypic response but also normalizes FBG and insulin levels in mice that continue to gain weight.

Our work strengthens the theory that metabolic status is an important regulator for the development of cardiac disease, suggesting that obesity might exacerbate the response to pressure overload by provoking distinct signaling mechanisms. We also think that JNK1/2 represents a particularly interesting signaling cascade in the heart because it impacts remodeling due to metabolic changes and inflammation. The downstream transcription factor of JNK1/2, c-Jun, is thought to regulate expression of the IGF pathway in the heart, providing a critical link between metabolism and hypertrophic growth (Sundaresan et al. 2012; Webster 2012). c-Jun is also differentially expressed in HF and metabolic disease, which our data confirm. Previous work has demonstrated that inhibition of JNK1/2 signaling or translocation to mitochondria reduces myocardial injury following ischemia–reperfusion (Kaiser et al. 2005; Chambers et al. 2013), suggesting the importance of JNK1/2 for transducing ischemic signals in the heart. In addition, other work has shown the importance of JNK inflammation-mediated cardiac dysfunction, as inhibition of JNK signaling prevents functional and metabolic changes due to the strong proinflammatory stimulus, lipopolysaccharide (LPS) (Chokshi et al. 2012). Thus, JNK1/2 signaling could be integrating signaling responses to various stimuli, leading to synergistic results.

### Electrophysiological remodeling associated with metabolic condition

Given the hypertrophic and mechanical responses to TAC with DIO, one might expect that EP alterations would demonstrate similarly exacerbated changes. Interestingly, TAC did not significantly impact EP properties on a DIO background. Both CV and APD80 restitution curves were similar for DIO sham and DIO TAC hearts, which suggests that metabolic state is an important regulator of cardiac EP remodeling. These results are particularly interesting when considering remodeling in HF models. Likewise to hypertrophic remodeling studies, EP experiments are typically conducted using normal weight animals. Therefore, many EP effects demonstrated with hypertrophy and HF may be significantly impacted by comorbid metabolic conditions.

In chow hearts TAC significantly reduced CV, which agrees with previously published findings (Akar et al. 2004, 2007; Poelzing and Rosenbaum 2004; Glukhov et al. 2012), and CV slowing in the chow TAC myocardium could promote arrhythmia susceptibility. Although overall CVs trend toward reduction in DIO compared with chow sham hearts, TAC did not slow CV of DIO hearts. Restitution curves for DIO sham and TAC hearts completely overlap, suggesting preserved CV in DIO TAC hearts. Likewise to CV restitution, DIO sham and TAC APD80 restitution curves were superimposed, and APD80 restitution curves were also similar for chow sham and TAC hearts.

These EP results may also be interesting in the context of the obesity paradox, which describes the phenomenon that obese patients with HF have improved mortality (Lavie et al. 2003). Despite the clear association between obesity and the development of HF, the inverse relationship between body mass index (BMI) and mortality in patients with HF has been consistently documented (Kenchaiah et al. 2002; Artham et al. 2008). Cardiovascular mortality is reduced in HF with concomitant BMI elevation (Oreopoulos et al. 2008). For overweight and obese patients, the reductions in cardiovascular mortality are 19% and 40%, respectively. This survival benefit of BMI correlates well with measures of percent body fat, with patients in the greatest quintile of percent body fat and BMI having the greatest event-free survival. Obesity is thought to produce a “metabolic reserve” in chronic heart disease (Lavie et al. 2003); however, the mechanism for such reserve has not been elucidated. Because obesity in our model regulates arrhythmogenic remodeling in hypertrophy, metabolic remodeling or reserves could potentially be protective against lethal arrhythmias.

The precedent for the obesity paradox in relationship to arrhythmia outcomes has been demonstrated for atrial fibrillation. Cardiovascular mortality and cumulative adverse events were reduced in overweight and obese relative to normal weight patients, and Badheka et al. (2010) speculate several mechanisms for the obesity paradox in atrial fibrillation. Notably, activation of the renin–angiotensin system is associated with electrical remodeling in atrial fibrillation (Novo et al. 2008). However, obese patients have lower plasma renin and norepinephrine levels compared with lean patients during treadmill testing (Weber et al. 2001). Such a mechanism could also impact electrical remodeling in HF.

## Limitations

For this work, we only conducted studies in WT mice and have not pursued investigations in genetically modified animals. However, based on selective activation of the JNK1/2 signaling pathway, we suspect that inhibition of this pathway will blunt the hypertrophic response from DIO and TAC. Our findings may explain why studies have shown contradicting results when MKK4, the upstream JNK1/2 activator, signaling is reduced. Activation of the JNK1/2 pathway may only be prohypertrophic under certain baseline metabolic conditions. However, to strengthen the molecular underpinnings of cardiomyocyte hypertrophy due to pressure overload and obesity, further studies using genetically modified animals will be needed. In addition, we conducted kinase activation and inflammatory response studies at 1 week after TAC, which previous work has suggested should be peak activation (Xia et al. 2009; Nagai et al. 2011). However, we cannot rule out earlier or later activation of additional pathways. Also, in tackling the challenge of starting from a dramatic phenotypic response to deducing the responsible underlying mechanism, we necessarily had to limit the number of signaling pathways screened. Thus, other pathways could be involved in the hypertrophic response to DIO and TAC. We also did not conduct blood pressure measurements, which could be viewed as a limitation of this study. While elevation in blood pressure should not impact the afterload on the heart following TAC, as this would be small relative to TAC pressure. However, if the DIO mice had hypertension prior to surgery, as can be true with obesity (Surwit et al. 1988; Mills et al. 1993), it is possible that the hearts from DIO animals were impacted by baseline blood pressure elevation. Thus, the effect we observe could be related to sequela of obesity, rather than obesity itself. Importantly, we did not observe that the output measures were dependent on presurgical BW, suggesting baseline body habitus did not regulate the hypertrophic response; nor did we observe significant remodeling in the DIO sham hearts, indicating that the afterload in mice with elevated BW was not significant enough to induce hypertrophy.

## Conclusions

Our model of cardiac hypertrophy and failure, in which DIO is produced prior to TAC more accurately recapitulates the HF progression in humans, as the majority of heart disease occurs on a longstanding background of obesity and metabolic disease. In addition, the degree of pressure overload utilized in these experiments was insufficient to induce cardiac hypertrophy and dysfunction in the animals fed on the standard chow diet, indicating that we have induced significant remodeling with moderate pressure overload, which may better mimic human hypertension. As this model more closely reflects the circumstances of cardiovascular disease in human, the mechanisms of remodeling, such as JNK1/2 activation, might be more reflective of HF pathogenesis. DIO could also be combined with a variety of other hypertrophic stimuli to induce cardiovascular remodeling, such as gene engineering or tachypacing, and may significantly modulate the response to these conditions. Similar DIO animal models may also be highly relevant for the numerous other disorders, not limited to cardiovascular disease, that occur in the setting of comorbid obesity.

## References

[b1] Akar FG, Spragg DD, Tunin RS, Kass DA, Tomaselli GF (2004). Mechanisms underlying conduction slowing and arrhythmogenesis in nonischemic dilated cardiomyopathy. Circ. Res.

[b2] Akar FG, Nass RD, Hahn S, Cingolani E, Shah M, Hesketh GG (2007). Dynamic changes in conduction velocity and gap junction properties during development of pacing-induced heart failure. Am. J. Physiol. Heart Circ. Physiol.

[b3] Artham SM, Lavie CJ, Patel HM, Ventura HO (2008). Impact of obesity on the risk of heart failure and its prognosis. J. Cardiometab. Syndr.

[b4] Auger-Messier M, Accornero F, Goonasekera SA, Bueno OF, Lorenz JN, van Berlo JH (2013). Unrestrained p38 MAPK activation in dusp1/4 double-null mice induces cardiomyopathy. Circ. Res.

[b5] Badheka AO, Rathod A, Kizilbash MA, Garg N, Mohamad T, Afonso L (2010). Influence of obesity on outcomes in atrial fibrillation: yet another obesity paradox. Am. J. Med.

[b6] Chambers JW, Pachori A, Howard S, Iqbal S, LoGrasso PV (2013). Inhibition of JNK mitochondrial localization and signaling is protective against ischemia/reperfusion injury in rats. J. Biol. Chem.

[b7] Chess DJ, Stanley WC (2008). Role of diet and fuel overabundance in the development and progression of heart failure. Cardiovasc. Res.

[b8] Chess DJ, Lei B, Hoit BD, Azimzadeh AM, Stanley WC (2007). Deleterious effects of sugar and protective effects of starch on cardiac remodeling, contractile dysfunction, and mortality in response to pressure overload. Am. J. Physiol. Heart Circ. Physiol.

[b9] Chess DJ, Lei B, Hoit BD, Azimzadeh AM, Stanley WC (2008). Effects of a high saturated fat diet on cardiac hypertrophy and dysfunction in response to pressure overload. J. Card. Fail.

[b10] Chokshi A, Drosatos K, Cheema FH, Ji R, Khawaja T, Yu S (2012). Ventricular assist device implantation corrects myocardial lipotoxicity, reverses insulin resistance, and normalizes cardiac metabolism in patients with advanced heart failure. Circulation.

[b11] Choukroun G, Hajjar R, Fry S, del Monte F, Haq S, Guerrero JL (1999). Regulation of cardiac hypertrophy in vivo by the stress-activated protein kinases/c-Jun NH2-terminal kinases. J. Clin. Invest.

[b12] Chu C-H, Tzang B-S, Chen L-M, Liu C-J, Tsai F-J, Tsai C-H (2009). Activation of insulin-like growth factor II receptor induces mitochondrial-dependent apoptosis through G*α*q and downstream calcineurin signaling in myocardial cells. Endocrinology.

[b13] DeBosch B, Treskov I, Lupu TS, Weinheimer C, Kovacs A, Courtois M (2006). Akt1 is required for physiological cardiac growth. Circulation.

[b14] Dou Y, Arlock P, Arner A (2007). Blebbistatin specifically inhibits actin-myosin interaction in mouse cardiac muscle. Am. J. Physiol. Cell Physiol.

[b15] Duda MK, O’Shea KM, Lei B, Barrows BR, Azimzadeh AM, McElfresh TE (2008). Low-carbohydrate/high-fat diet attenuates pressure overload-induced ventricular remodeling and dysfunction. J. Card. Fail.

[b16] Esposito G, Prasad SVN, Rapacciuolo A, Mao L, Koch WJ, Rockman HA (2001). Cardiac overexpression of a Gq inhibitor blocks induction of extracellular signal–regulated kinase and c-Jun NH2-terminal kinase activity in in vivo pressure overload. Circulation.

[b17] Felker GM, Fiuzat M, Shaw LK, Clare R, Whellan DJ, Bettari L (2012). Galectin-3 in ambulatory patients with heart failure results from the HF-ACTION study. Circulation. Heart failure.

[b18] Glukhov AV, Flagg TP, Fedorov VV, Efimov IR, Nichols CG (2010). Differential K(ATP) channel pharmacology in intact mouse heart. J. Mol. Cell. Cardiol.

[b19] Glukhov AV, Fedorov VV, Kalish PW, Ravikumar VK, Lou Q, Janks D (2012). Conduction remodeling in human end-stage non-ischemic left ventricular cardiomyopathy. Circulation.

[b20] Guh DP, Zhang W, Bansback N, Amarsi Z, Birmingham CL, Anis AH (2009). The incidence of co-morbidities related to obesity and overweight: a systematic review and meta-analysis. BMC Public Health.

[b21] Han MS, Jung DY, Morel C, Lakhani SA, Kim JK, Flavell RA (2013). JNK expression by macrophages promotes obesity-induced insulin resistance and inflammation. Science.

[b22] Hasenfuss G (1998). Animal models of human cardiovascular disease, heart failure and hypertrophy. Cardiovasc. Res.

[b23] Hill AB (1965). The environment and disease: association or causation?. Proc. R. Soc. Med.

[b24] Hirosumi J, Tuncman G, Chang L, Görgün CZ, Uysal KT, Maeda K (2002). A central role for JNK in obesity and insulin resistance. Nature.

[b25] Houser SR, Margulies KB, Murphy AM, Spinale FG, Francis GS, Prabhu SD (2012). Animal models of heart failure: a scientific statement from the American Heart Association. Circ. Res.

[b26] Jagasia D, Whiting JM, Concato J, Pfau S, McNulty PH (2001). Effect of non–insulin-dependent diabetes mellitus on myocardial insulin responsiveness in patients with ischemic heart disease. Circulation.

[b27] Kaiser RA, Liang QR, Bueno O, Huang Y, Lackey T, Klevitsky R (2005). Genetic inhibition or activation of JNK1/2 protects the myocardium from ischemia-reperfusion-induced cell death in vivo. J. Biol. Chem.

[b28] Kemi OJ, Ceci M, Wisloff U, Grimaldi S, Gallo P, Smith GL (2008). Activation or inactivation of cardiac Akt/mTOR signaling diverges physiological from pathological hypertrophy. J. Cell. Physiol.

[b29] Kenchaiah S, Evans JC, Levy D, Wilson PW, Benjamin EJ, Larson MG (2002). Obesity and the risk of heart failure. N. Engl. J. Med.

[b30] Kress AM, Hartzel MC, Peterson MR (2005). Burden of disease associated with overweight and obesity among U.S. military retirees and their dependents, aged 38-64, 2003. Prev. Med.

[b31] Lang D, Glukhov AV, Efimova T, Efimov IR (2011). Role of Pyk2 in cardiac arrhythmogenesis. Am. J. Physiol. Heart Circ. Physiol.

[b32] Laughner JI, Ng FS, Sulkin MS, Arthur RM, Efimov IR (2012). Processing and analysis of cardiac optical mapping data obtained with potentiometric dyes. Am. J. Physiol. Heart Circ. Physiol.

[b33] Lavie CJ, Osman AF, Milani RV, Mehra MR (2003). Body composition and prognosis in chronic systolic heart failure: the obesity paradox. Am. J. Cardiol.

[b34] Liu W, Zi M, Jin J, Prehar S, Oceandy D, Kimura TE (2009). Cardiac-specific deletion of Mkk4 reveals its role in pathological hypertrophic remodeling but not in physiological cardiac growth. Circ. Res.

[b35] Lopaschuk GD, Kelly DP (2008). Signalling in cardiac metabolism. Cardiovasc. Res.

[b36] Mandavia CH, Pulakat L, DeMarco V, Sowers JR (2012). Over-nutrition and metabolic cardiomyopathy. Metabolism.

[b37] Marber MS, Rose B, Wang Y (2011). The p38 mitogen-activated protein kinase pathway–a potential target for intervention in infarction, hypertrophy, and heart failure. J. Mol. Cell. Cardiol.

[b38] McMullen JR, Shioi T, Zhang L, Tarnavski O, Sherwood MC, Kang PM (2003). Phosphoinositide 3-kinase (p110*α*) plays a critical role for the induction of physiological, but not pathological, cardiac hypertrophy. Proc. Natl Acad. Sci.

[b39] Mills E, Kuhn CM, Feinglos MN, Surwit R (1993). Hypertension in CB57BL/6J mouse model of non-insulin-dependent diabetes mellitus. Am. J. Physiol.

[b40] Mosterd A, Hoes AW (2007). Clinical epidemiology of heart failure. Heart.

[b41] Nagai T, Anzai T, Kaneko H, Mano Y, Anzai A, Maekawa Y (2011). C-reactive protein overexpression exacerbates pressure overload-induced cardiac remodeling through enhanced inflammatory response. Hypertension.

[b42] Novo G, Guttilla D, Fazio G, Cooper D, Novo S (2008). The role of the renin–angiotensin system in atrial fibrillation and the therapeutic effects of ACE-Is and ARBS. Br. J. Clin. Pharmacol.

[b43] Oreopoulos A, Padwal R, Kalantar-Zadeh K, Fonarow GC, Norris CM, McAlister FA (2008). Body mass index and mortality in heart failure: a meta-analysis. Am. Heart J.

[b44] Poelzing S, Rosenbaum DS (2004). Altered connexin43 expression produces arrhythmia substrate in heart failure. Am. J. Physiol. Heart Circ. Physiol.

[b45] Raher MJ, Thibault HB, Buys ES, Kuruppu D, Shimizu N, Brownell AL (2008). A short duration of high-fat diet induces insulin resistance and predisposes to adverse left ventricular remodeling after pressure overload. Am. J. Physiol. Heart Circ. Physiol.

[b46] Rockman HA, Ross RS, Harris AN, Knowlton KU, Steinhelper ME, Field LJ (1991). Segregation of atrial-specific and inducible expression of an atrial natriuretic factor transgene in an in vivo murine model of cardiac hypertrophy. Proc. Natl Acad. Sci. USA.

[b47] Rockman HA, Ono S, Ross RS, Jones LR, Karimi M, Bhargava V (1994). Molecular and physiological alterations in murine ventricular dysfunction. Proc. Natl Acad. Sci. USA.

[b48] Rose BA, Force T, Wang Y (2010). Mitogen-activated protein kinase signaling in the heart: angels versus demons in a heart-breaking tale. Physiol. Rev.

[b49] Shimizu I, Minamino T, Toko H, Okada S, Ikeda H, Yasuda N (2010). Excessive cardiac insulin signaling exacerbates systolic dysfunction induced by pressure overload in rodents. J. Clin. Investig.

[b50] Shiojima I, Yefremashvili M, Luo Z, Kureishi Y, Takahashi A, Tao J (2002). Akt signaling mediates postnatal heart growth in response to insulin and nutritional status. J. Biol. Chem.

[b51] Sopontammarak S, Aliharoob A, Ocampo C, Arcilla RA, Gupta MP, Gupta M (2005). Mitogen-activated protein kinases (p38 and c-Jun NH2-terminal kinase) are differentially regulated during cardiac volume and pressure overload hypertrophy. Cell Biochem. Biophys.

[b52] Steinbusch LK, Luiken JJ, Vlasblom R, Chabowski A, Hoebers NT, Coumans WA (2011). Absence of fatty acid transporter CD36 protects against Western-type diet-related cardiac dysfunction following pressure overload in mice. Am. J. Physiol. Endocrinol. Metab.

[b53] Sundaresan NR, Vasudevan P, Zhong L, Kim G, Samant S, Parekh V (2012). The sirtuin SIRT6 blocks IGF-Akt signaling and development of cardiac hypertrophy by targeting c-Jun. Nat. Med.

[b54] Surwit RS, Kuhn CM, Cochrane C, McCubbin JA, Feinglos MN (1988). Diet-induced type II diabetes in C57BL/6J mice. Diabetes.

[b55] Tuncman G, Hirosumi J, Solinas G, Chang L, Karin M, Hotamisligil GS (2006). Functional in vivo interactions between JNK1 and JNK2 isoforms in obesity and insulin resistance. Proc. Natl Acad. Sci.

[b56] Walsh K (2006). Akt signaling and growth of the heart. Circulation.

[b57] Wang Y, Su B, Sah VP, Brown JH, Han J, Chien KR (1998). Cardiac hypertrophy induced by mitogen-activated protein kinase kinase 7, a specific activator for c-Jun NH2-terminal kinase in ventricular muscle cells. J. Biol. Chem.

[b58] Weber MA, Neutel JM, Smith DH (2001). Contrasting clinical properties and exercise responses in obese and lean hypertensive patients. J. Am. Coll. Cardiol.

[b59] Webster KA (2012). A sirtuin link between metabolism and heart disease. Nat. Med.

[b60] Wilkins BJ, Dai YS, Bueno OF, Parsons SA, Xu J, Plank DM (2004). Calcineurin/NFAT coupling participates in pathological, but not physiological, cardiac hypertrophy. Circ. Res.

[b61] Xia Y, Lee K, Li N, Corbett D, Mendoza L, Frangogiannis NG (2009). Characterization of the inflammatory and fibrotic response in a mouse model of cardiac pressure overload. Histochem. Cell Biol.

